# Retinal Inflammation, Cell Death and Inherited Retinal Dystrophies

**DOI:** 10.3390/ijms22042096

**Published:** 2021-02-20

**Authors:** Lorena Olivares-González, Sheyla Velasco, Isabel Campillo, Regina Rodrigo

**Affiliations:** 1 Pathophysiology and Therapies for Vision Disorders, Principe Felipe Research Center (CIPF), Eduardo Primo Yúfera 3, 46012 Valencia, Spain; lolivares@cipf.es (L.O.-G.); svelasco@cipf.es (S.V.); icampillo@cipf.es (I.C.); 2 Joint Research Unit on Rare Diseases CIPF—Health Research Institute Hospital La Fe, IIS-La Fe, 46026 Valencia, Spain; 3 CIBER de Enfermedades Raras (CIBERER), 28029 Madrid, Spain

**Keywords:** retinal dystrophies, cell death, inflammation, TNFα

## Abstract

Inherited retinal dystrophies (IRDs) are a group of retinal disorders that cause progressive and severe loss of vision because of retinal cell death, mainly photoreceptor cells. IRDs include retinitis pigmentosa (RP), the most common IRD. IRDs present a genetic and clinical heterogeneity that makes it difficult to achieve proper treatment. The progression of IRDs is influenced, among other factors, by the activation of the immune cells (microglia, macrophages, etc.) and the release of inflammatory molecules such as chemokines and cytokines. Upregulation of tumor necrosis factor alpha (TNFα), a pro-inflammatory cytokine, is found in IRDs. This cytokine may influence photoreceptor cell death. Different cell death mechanisms are proposed, including apoptosis, necroptosis, pyroptosis, autophagy, excessive activation of calpains, or parthanatos for photoreceptor cell death. Some of these cell death mechanisms are linked to TNFα upregulation and inflammation. Therapeutic approaches that reduce retinal inflammation have emerged as useful therapies for slowing down the progression of IRDs. We focused this review on the relationship between retinal inflammation and the different cell death mechanisms involved in RP. We also reviewed the main anti-inflammatory therapies for the treatment of IRDs.

## 1. Introduction

Inherited retinal dystrophies (IRDs) constitute a heterogeneous group of retinal diseases that cause the progressive loss of vision. IRDs affect about 1 in 2000 to 3000 individuals [1]. IRDs are mainly associated with photoreceptor (rods and cones) dysfunction and loss that eventually leads to blindness. Retinal pigment epithelium (RPE) cells are also associated with IRDs (e.g., RPE65 gene mutations). Cell death may be exacerbated by the accumulation of reactive oxygen species (ROS) and inflammation [2]. IRDs can be subdivided, depending on the photoreceptor affected, into rod-dominated dystrophies, cone- dominated dystrophies, and generalized dystrophies involving both rods and cones [3]. IRDs present high genetic and clinical heterogeneity. More than 300 genes with autosomal dominant and recessive, X-linked, or mitochondrial inheritance patterns are linked to this group of diseases [4]. Moreover, different mutations in one gene can result in different phenotypes. IRDs include syndromic forms such as Usher syndrome and non-syndromic forms such as Retinitis Pigmentosa (RP), Leber’s congenital amaurosis, Stargardt’s macular dystrophy, choroideremia, macular degeneration, or congenital stationary night blindness. This heterogeneity makes difficult to find the specific mutation, as well as the correct treatment for most of these diseases.

RP is the most common IRD, with a prevalence of approximately 1 in 4000 individuals [5]. Patients with RP typically lose night vision in adolescence, peripheral vision in young adulthood, and central vision later in life. At the cellular level, RP is characterized by a progressive degeneration of rod photoreceptors at early stages due to genetic mutations. Rod degeneration causes the release of inflammatory molecules—free radicals, among others—that cause a hostile microenvironment that eventually affects the cones’ survival [6]. The purpose of this review was to provide an overview of the inflammatory processes and some cell death mechanisms involved in RP, as well as the current anti-inflammatory approaches for slowing down the photoreceptor cell loss.

## 2. Retinal Inflammation in IRDs

### 2.1. Ocular Inflammation

Ocular inflammation is a common cause of visual impairment. Inflammation is the immune system’s response to a harmful stimulus that is caused by different factors, mainly pathogens, cell damage, toxic metabolites, and even genetic stressors. It is a defense mechanism to recover tissue homeostasis [7]. The stimuli can induce acute inflammation (short period) or chronic inflammation (extended period), activating different signaling pathways. During acute inflammatory responses, the damage is minimized or eliminated. However, acute, uncontrolled, and excessive inflammation may become chronic and harmful to cells [7,8,9].

Neuroinflammation is the inflammation of the nervous tissue. It is considered a chronic inflammation with sustained activation of glial cells (microglia, astrocytes) and recruitment of other immune cells into the nervous tissue (brain, spinal cord, or retina) [10]. Microglia cells, the resident innate immune cells in the retina and other nervous tissue, are potential cellular regulators of inflammation [11]. They perform immune surveillance and they are activated upon pathologic signals. Under normal conditions or in the absence of damage, microglia cells have a branched morphology with a small round soma, and participate in processes of axonal growth, synaptic remodeling, and neuronal survival. In response to harmful stimuli, tissue damage, or free radicals, microglia cells move to a reactive state, which is characterized by the acquisition of an amoeboid morphology, acquiring the characteristic functions of macrophages such as phagocytosis, induction of the inflammatory process, and presentation of antigens to lymphocytes [9,12,13,14]. Activated microglia and macrophages secrete pro-inflammatory mediators (cytokines, chemokines, etc.) and upregulate the expression of inducible nitric oxide synthase (iNOS) that act on other cells (e.g., photoreceptor cells) [15]. The nuclear factor kappa beta (NF-kB), mitogen-activated protein kinase (MAPK), or Janus kinase/signal transducer and activator transcription (JAK-STAT) signaling pathways are the most common pathways activated after a harmful stimulus. These signaling pathways promote the production of different inflammatory mediators such as the pro-inflammatory interleukins (ILs) IL1-β, IL-6, IL-8, and IL-12; tumor necrosis factor alpha (TNFα); gamma interferon (IFN-γ); granulocyte monocyte colony-stimulating factor (GM-CSF); anti-inflammatory cytokines (IL-4, IL-10, IL-11), transforming growth factor beta (TGF-β); and inflammatory proteins such as C-reactive protein (CPR), haptoglobulin, serum amyloid protein, fibrinogen, and alpha-1 acid glycoprotein.

### 2.2. Inflammation in RP

#### 2.2.1. Local Inflammation

In the retina, the chronic inflammation or neuroinflammation causes neuronal damage and leads to the development or progression of a variety of neurodegenerative diseases including IRDs, retinal neovascular disease [16], diabetic retinopathy (DR), or age-related macular degeneration (AMD) [17]. In these degenerative retinal diseases, there is specific damage at the onset of each disease, but studies suggest that chronic and low-grade inflammation is also involved in their progression.

**Figure 1:**
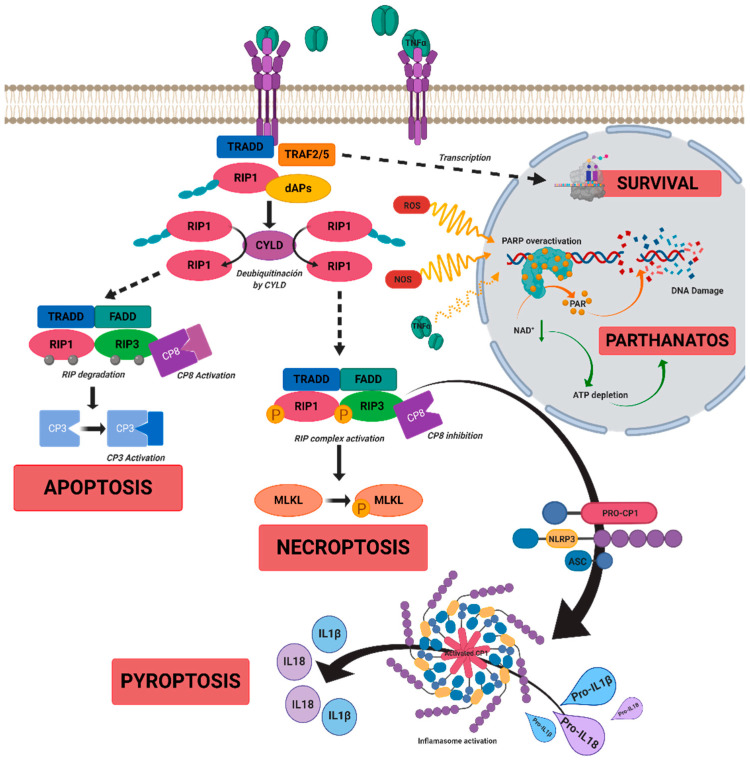
A scheme of possible cell death mechanisms during of tumor necrosis factor alpha (TNFα)-induced signaling in inherited retinal dystrophies (IRDs). TNFα can simultaneously activate multiple signaling pathways of cell death or survival. TNFα binds to tumor necrosis factor receptor 1 (TNFR1), triggering three functional states. (i) The intracellular domain of TNFR1 recruits a death-domain containing adaptor protein (TRADD). TRADD recruits TNF receptor-associated factor 2 (TRAF2) and receptor-interacting protein kinase 1 (RIPK1) to form Complex 1. Complex 1 seems to be important for nuclear factor kappa beta (NF-κB)activation. NF-κB regulates anti-apoptotic genes to block the initiation of apoptosis by Complex 2. (ii) Complex 1 dissociates from TNFR1 and integrates Fas-associated protein with death domain (FADD) and pro-caspase 8 to form Complex 2. The FADD/caspase 8 association depends on complexes containing unubiquitinated RIPK1 as a scaffold. Activated caspase 8 induces caspase 3 and apoptosis. Under apoptotic conditions, active caspase 8 prevents further necroptotic signaling by cleaving and inactivating RIPK1 and RIPK3. (iii) Necroptosis is also mediated through TNFα signaling, when caspase 8 is not active. RIPK1 recruits RIPK3 to form the necrosome complex. RIPK3 phosphorylates the pseudokinase kinase-like domain of mixed-lineage kinase do-main-like (MLKL), leading to its oligomerization. Thus, MLKL recruitment to the plasma membrane induces necroptosis by triggering Ca^+^ and Na^2+^ influx into the cell. RIPK3 can also promote the nucleotide-binding oligomerization domain containing protein (NOD)-like receptor (NLR) family protein 3 (NLRP3) inflammasome and interleukin (IL)-1β inflammatory responses. TNFα or oxidative stress could activate parthanatos through the overactivation of poly [ADP-ribose] polymerase 1 (PARP1). PARP1 cleaves nicotinamide adenine dinucleotide (NAD+) to nicotinamide and adenosine diphosphate (ADP-ribose). PARPs couple one or more ADP-ribose (PAR) to acceptor proteins (PARylation) and components of the DNA repair machinery. Overactivation of PARP1 can deplete cellular adenosine triphosphate (ATP) and NAD+ storage and lead to bioenergetic collapse and cell death. Adapted from Olivares-González et al. [28].

In RP, sustained and chronic inflammation is observed in parallel with the progression of the disease in patients and *rd10* mice, a murine model of autosomal recessive RP [18,19]. Evidence of inflammatory cell infiltration, including microglia, macrophages, natural killer cells, and monocytes has been reported in the vitreous humor and in the retina of patients with RP [20,21,22]. Microglia activation and upregulation of inflammatory mediators such as TNFα, IL-6, IL1α, IL1β, monocyte chemoattractant proteins 1 and 2 (MCP-1, MCP-2), or platelet growth factor (PGF) are present in both patients and murine models of RP at early stages of the disease, and they are postulated to contribute to photoreceptor death [23,24,25]. Activated macrophages and glial cells release the pro-inflammatory cytokine TNFα, which participates in the induction and maintenance of the immune response. It may be involved in both cell death and survival pathways (see Figure 1) [26]. Moreover, TNFα is a negative regulator of the transcription factor orthodenticle homeobox 2 (OTX2) expressed in RPE that is critical for the outer retina maintenance [27]. Upregulation of TNFα is found in several inflammatory eye diseases including RP, glaucoma, uveitis, retinal vascular tumors, Adamantiades–Behcet disease, diabetic macular edema, DR, or AMD [24,28,29,30,31,32,33,34,35,36]. In particular, elevated TNFα content is present in the aqueous humor of patients and in the retina of murine models of RP [37,38,39,40]. The blockade of TNFα expression in T17M mice with a rhodopsin (RHO) mutation (a model of RP) or the use of antibodies against TNFα (e.g., adalimumab) in *rd10* mice slow down photoreceptor death [28,38,41], suggesting that it may be an attractive therapeutic target.

#### 2.2.2. Peripheral Inflammation

The eye has an immune privilege to limit the extent of immune responses and to preserve vision [42]. Ocular fluids and cells have soluble and cell-bound inhibitory factors and immunosuppressive factors including TGF-β2, retinoic acid, galectins, etc. to maintain the immune privilege [15]. Recent studies show not only local ocular inflammation but also peripheral inflammatory responses in RP patients with high serum IL8, regulated upon activation, normal T cell expressed, and secreted chemokine (C-C motif) ligand 5 (CL5), and CPR levels that correlate with an impairment of the visual function [18,25]. Therefore, systemic inflammation could also contribute to the progression of RP [18]. Moreover, studies in animal models suggest that peripheral damage, such as a systemic infection or a chronic inflammatory process, may also play a role in retinal degeneration in patients with IRDs. Thus, this should be taken into account in the development of possible treatments [43].

### 2.3. Inflammation and Cell Death in RP

Inflammation and cell death are interconnected. Studies propose that inflammation is a consequence of the photoreceptor degeneration triggered by the genetic defects. Nevertheless, other studies suggest that inflammatory cells promote retinal degeneration by their cytotoxic effects on photoreceptor cells [44]. Therefore, it is difficult to discern the cause–effect relationship in RP and other IRDs. During photoreceptor cell death, inflammatory cells are activated by releasing inflammatory substances that contribute to rod degeneration and eventually to cone cell death and loss of central vision [17,44,45]. On the other hand, degenerated cells secrete intracellular molecules like adenosine triphosphate (ATP), S100 proteins, high mobility group box 1 (HMGB1), RNA, etc., called damage-associated molecular patterns (DAMPs). DAMPs are recognized by specialized innate immune receptors including Toll-like receptors (TLRs) and nucleotide-binding oligomerization domain containing protein (NOD)-like receptors (NLRs) that induce “sterile inflammation” [46]. The release of DAMPs seems to be an important link between cell death and inflammation in IRDs and other retinal diseases including DR or AMD [15]. ATP, which acts as a neuro- and gliotransmitter in the retina, is secreted or released by apoptotic or necrotic cells, respectively, recruiting innate immune cells (macrophages, microglia) to the damage site. In particular, the binding of extracellular ATP by P2X7, a purinergic receptor, on phagocytes (macrophages, microglia, etc.) leads to chemokine release through the activation of the protein kinase C (PKC)/MAP kinase pathway and to the release of the pro-inflammatory cytokines IL-6 and TNFα [47]. Overactivation of P2X7 has a detrimental role in IRDs and other retinal diseases such as DR, AMD or glaucoma [48]. Besides, extracellular ATP stimulates the formation of inflammasomes, large intracellular multiproteic complexes that are key players during the innate immune response [49,50]. Apart from ATP, inflammasomes respond to other DAMPs and danger signals from pathogens. The inflammasomes consist of sensor proteins called NLR family proteins (NLRPs), an adaptor protein called apoptosis speck-like protein (ASC), and caspase 1. Canonical inflammasomes, assembled by the (nucleotide-binding oligomerization domain containing protein (NOD)-like receptor (NLR) family protein 2 (NLRP1), NLRP3 and Aim2 sensor proteins, activate caspase 1, promoting the maturation of the cytokines IL1β and IL18. This leads to the infiltration of more immune cells and results in the generation and maintenance of the inflammatory state, and ultimately to pyroptotic cell death [15,51]. Activation or upregulation of the inflammatory components of the NLRP3 inflammasome (protein NLRP3, apoptosis speck-like protein (ASC), and caspase 1) or of the pro-inflammatory cytokines IL1β and IL18 is observed in canine and murine models of RP. This upregulation can be involved in the death of photoreceptors by pyroptosis [23,28,52,53].

## 3. Cell Death Mechanisms in IRDs

In RP, early studies considered classical apoptosis as the main cell death mechanism responsible for rod cell death [54]. However, secondary cone cell death remained unclear [55]. Different anti-apoptotic strategies based on caspase inhibition failed to reduce photoreceptor cell death in animal models of RP. Therefore, other non-apoptotic mechanisms were proposed to participate in this degenerative process, including regulated necrosis (necroptosis, pyroptosis, ferroptosis, parthanatos), autophagy, calpain activation, or cyclic guanosine monophosphate (cGMP)-dependent photoreceptor death [26,56,57,58,59]. Indeed, several studies suggested that these alternative cell death mechanisms may be the dominant modes of cell death in RP [60]. In this review, we focused on cell death mechanisms that are related to TNFα, a potent regulator of both apoptotic and necrotic pathways (Figure 1).

### 3.1. Apoptosis

Apoptosis is a process of programmed cell death, which is accompanied by a reduction in cell volume, chromatin condensation, and nuclear fragmentation. During apoptosis, there is no release of cell content. It is dependent on the activation of a series of cysteine proteases called caspases [61,62]. Cellular damage produces the activation of the initiating caspases (8 and 9), which activate the effector caspases (3, 6 and 7). Effector caspases initiate a cascade of events that result in deoxyribonucleic acid (DNA) fragmentation, endonuclease activation, the destruction of nuclear and cytoskeleton proteins, and ligand expression to attract phagocytic cells. It is a highly conserved and genetically controlled process. It is activated by the cell itself when it detects damage, which is known as the intrinsic pathway of apoptosis. In the case of activation by an interaction with immune system cells, it is called the extrinsic pathway of apoptosis. The intrinsic pathway is characterized by the permeabilization of the mitochondrial membrane, which leads to the release of the cytoplasm of pro-apoptotic factors causing the activation of caspase 9, and the consequent activation of caspase 3 [62,63]. The extrinsic pathway is activated through members of the TNF receptor family, also called death receptors. TNFα or Fas produced by immune cells binds to death receptors on the surface of damaged cells. The most characteristic death receptor is Fas (, which activates caspase 8. Caspase 8 initiates an activation cascade that includes caspases 3, 6, and 7, executing the death signal [61,63].

Apoptotic photoreceptor and caspase 3 activation have been reported in models of RP including *rds*, *rd1*, and *rd10* mice or S334ter rats [64,65,66,67]. In these RP models, caspase 3 activation correlates with rapid photoreceptor degeneration, which was partially or transient rescued by caspase 3 inhibitors or by ablation of caspase 3 [68,69].

### 3.2. Necrosis

Necrosis is a passive and uncontrolled process of cell death, induced by an external injury such as hypoxia or inflammation. It is accompanied by cellular swelling (oncosis), swelling of organelles, and early cell membrane rupture, leading to the release of intracellular content. This triggers an inflammatory cascade and tissue damage. Unlike apoptosis, necrosis is a process of energy-independent death, where the cell suffers severe damage that stops the cellular processes, and normally dies by oncosis and lysis [62,70]. Necrosis is very difficult or even impossible to block [59,70,71].

Other processes of programmed cell death (not apoptotic) are necroptosis, pyroptosis and parthanatos. In these processes, the cell death is genetically controlled and involves active cellular processes that can be blocked. Some authors classify necroptosis, pyroptosis, and parthanatos as types of regulated necrosis [72].

### 3.3. Necroptosis

Necroptosis is a type of regulated necrosis, whose activation is mediated by death receptors such as Fas or tumor necrosis factor receptor 1 (TNFR1) and the ligands TNFα or the apoptosis-inducing ligand (TRAIL). Multiple signals can potentially activate it, but TNFα seems to be the most important signal that triggers it under various pathological conditions. It is characterized by ATP decrease, plasma membrane rupture, and HMGB1 release, a DAMP molecule (see Section 2), among others. Activation of TNFR1 can trigger three functional states, depending on the set of regulatory proteins involved in the process (Figure 1). In particular, the receptor-interacting protein kinase 1 (RIPK1 or RIP1) activity plays an important role in promoting these states (survival, necroptosis, or apoptosis). (i) TNFR1 binds to RIPK1, and several ubiquitin ligases E3 to form the survival Complex I, where RIPK1 is polyubiquitinated, triggering cellular survival pathways. (ii) Activated RIPK1 interacts with Fas-associated protein with death domain (FADD) and caspase 8 to form Complex IIa, triggering apoptosis. (iii) Activated RIPK1 interacts with RIPK3 (or RIP3) to form Complex IIb that triggers necroptosis. The pre-necrotic RIPK1/RIPK3 complex promotes the recruitment, phosphorylation, and plasma membrane translocation of pseudokinase kinase-like domain of mixed-lineage kinase domain-like (MLKL) protein. Phosphorylated MLKL forms disulfide bond-dependent amyloid-like polymers and permeabilizes the plasma membrane, executing necroptosis [62,70,73,74]. In addition, RIPK3 can activate a series of signal transduction pathways inducing mitochondrial ROS production [61] or NLRP3 inflammasome activation and subsequent pyroptosis [75,76].

In the P23H-1 rhodopsin rat, upregulation of RIP1K/RIP3K complexes is associated with necroptosis in rod photoreceptors. The authors suggested that necroptotic rods can release their intracellular content, acting as DAMPs, which induce activation of NLRP3 inflammasome in cones and their subsequent cell death [52,77]. However, the main driver of rod cell death in the other rhodopsin rat model, the S334ter rat, is a caspase-dependent process, whereas cone cell death occurs through RIP3K-dependent necroptosis [55]. In *rd10* mice, an imbalance between RIP1K and RIP3K with a downregulation of RIP1K and an upregulation of RIP3K has observed at the early-intermediate stage (postnatal Day 23) of RP. At this age, some components of the NLRP3 inflammasome were increased [28]. Taken together, these results suggest that RIP3K upregulation would participate in NLRP3 inflammasome activation and subsequent photoreceptor cell death in *rd10* mice on this postnatal day. However, other authors did not observe changes in RIP1K or RIP3K at a similar age (postnatal Day 21). They proposed that apoptosis is responsible for rod degeneration and necroptosis mediates cone degeneration at later stages (Day 35 or later) in *rd10* mice [77].

### 3.4. Pyroptosis

Pyroptosis is a form of pro-inflammatory death that depends on pattern recognition receptors (PRRs) including TLRs and NLRs. These PPRs recognize pathogen-associated molecular patterns (PAMPs) or DAMPs (see Section 2). After stimulation by PAMPs or DAMPs, active caspase 1 catalyzes the passage of pro-IL1β and pro-IL-18 to their active forms, and the activation of the cytosolic protein gasdermin D (GSDMD) that, when cleaved, forms pores in the plasma membrane. This causes an ionic imbalance that facilitates the entry of water into the cell and ends up producing cellular lysis and the release of inflammatory cytokines to the extracellular environment [61,62,78]. During pyroptosis, the nuclear integrity is maintained, and condensation and fragmentation of the oligonucleosomal DNA are observed, but with a different pattern from that observed during apoptosis [79,80].

As described above, upregulation of some components of the NLRP3 inflammasome, including increase of the NLRP3 protein, caspase 1 activity, and IL1β gene expression, is observed in *rd10* retinas on postnatal Day 23 [28], and in other murine and canine models of RP (see Section 2). The high levels of TNFα and oxidative stress observed in models of RP could activate the NLRP3 inflammasome and precipitate pyroptosis.

### 3.5. Parthanatos

Parthanatos is a newly described form of cell death, dependent on the formation of poly- adenosine diphosphate ADP ribose (PAR) polymers, synthesized by poly [ADP-ribose] polymerase (PARP) enzymes. PARP enzymes are involved in several cellular processes including DNA repair, genomic stability, and cell death. The central PARP responsible for the formation of PAR polymers during DNA damage is PARP1. PARP1 activation triggers parthanatos due to this DNA damage, which can be caused by ROS, reactive nitrogen species (NOS), or even pro-inflammatory cytokines such as TNFα. The poly (ADP-ribosil)ation (or PARylation) of proteins, including auto-PARylation of PARP1, near the site of DNA damage facilitates the recruitment of effector proteins contributing to their repair. It is primarily a survival mechanism but excessive PARylation in damaged cells promotes parthanatos. Briefly, PARP1 activation results in PAR formation to mark the damaged site using nicotinamide adenine dinucleotide (NAD)+ and ATP, which leads to NAD+ depletion. PAR polymers induce apoptosis-inducing factor (AIF) translocation from the mitochondria to the cytoplasm. Although the mechanism is still unclear, NAD+ depletion may trigger depolarization of the mitochondrial membrane, facilitating AIF release. AIF interacts with the macrophage migration inhibitor factor (MIF) in the cytoplasm, which translocate to the nucleus and produces more DNA fragmentation, resulting in cell death [59,61,81].

PARP activation occurs in different models of RP including *rd10* mice, and P23H and S334ter rats (mutations in the RHO gene) [28,82]. The use of PARP inhibitors reduces PARylation and rod cell death in *rd1* mice [83].

## 4. Therapeutic Approaches to Reduce Inflammation and Cell Death

Currently, there are no effective treatments available to address the development and progression of IRDs, except for RPE65 gene therapy (Voretigene neparvovec-rzyl, Luxturna; Spark Therapeutics). Different therapeutic approaches are under research, including gene therapy, cell therapy, retinal prosthesis, or pharmacological therapy, depending on the stage of the disease. Pharmacological therapies are focused on slowing down the progression of IRDs and maintaining vision, mainly through neuroprotective, anti-apoptotic (or other cell death inhibition), antioxidant and anti-inflammatory mechanisms [84]. These therapies are independent of the genetic defect; however, they are not curative treatments, since they do not act on the genetic defect responsible for the disease.

### 4.1. Anti-Inflammatory Therapies

As mentioned above, inflammation and cell death are tightly interconnected. Therapeutic approaches for targeting inflammation may reduce photoreceptor cell death and help to maintain the retinal integrity for longer in IRDs. In particular, several strategies are aimed at preventing cone loss, a major problem for RP patients, because this leads to total blindness.

#### 4.1.1. Therapies against TNFα

TNFα seems to play an important role in the pathogenesis of inflammatory and neurodegenerative disorders including IRDs. Several TNFα inhibitors have been developed and approved for clinical use in inflammatory diseases such as rheumatoid arthritis, psoriasis, and ankylosing spondylitis. In ophthalmology, TNFα inhibitors, or biologics such as infliximab, etanercept, or adalimumab are used as off-label alternatives to “traditional” immunosuppressive and immune-modulatory treatments in non-infectious uveitis [85,86,87,88,89,90]. These biologics or their biosimilars are used because of their effectiveness to block TNFα binding to their receptors.

The use of anti-TNFα agents improves the survival of retinal cells in animal models of glaucoma, uveitis, DR, choroidal neovascularization, and RP [28,91,92,93]. In particular, intraperitoneal or intravitreal administration of adalimumab reduced photoreceptor cell death, PARP activity, and activation of the NLRP3 inflammasome and microglia at postnatal Day 18 or 23, respectively, in *rd10* mice [28,38]. TNFα inhibitors have been previously tested, although without therapeutic benefits, in patients with AMD or DR [88]. However, they have not been tested in patients with IRDs.

#### 4.1.2. Microglia Inhibition

Microglial cells play important roles in maintaining structure and proper retinal function. As previously described, they are the resident innate immune cells in the retina and potential cellular regulators of inflammation [11]. They perform immune surveillance. Pathologic signals activate them. Microglial activation is a hallmark of inflammation and it is observed in IRDs [94] and other retinal diseases such as AMD [95,96], RP [44,45], DR [97,98], or glaucoma [99]. Active microglia cells release neurotoxic and/or inflammatory mediators that exacerbate retinal cell death, such as TNFα, IL-1β, IL-6, and glutamate and increase iNOS expression [100]. In their activated state, they also secrete matrix metalloproteinase-9 (MMP-9), a type of protease that degrades the extracellular matrix and membrane proteins, resulting in the recruitment and activation of other immune cells. The activated microglia cells coexist in two phenotypes according to the environmental stimuli: pro-inflammatory (M1) or anti-inflammatory (M2). The polarization towards M1 forms during the progression of the disease and exacerbates the damage to photoreceptor cells in diseases such as DR [14,101].

Many therapeutic approaches are aimed at the inhibition of microglia activation to reduce the release of inflammatory substances (Table 1). Minocycline, a semi-synthetic tetracycline derivative, suppresses microglial activation by decreasing photoreceptor cell death, improving retinal structure and function in *rd10* mice [45]. In other retinal disorders like glaucoma or DR, minocycline also inhibits the release of the pro-inflammatory cytokines IL-1β, and TNF-α, the expression of PARP1, and photoreceptor cell death [102,103,104].

Resveratrol is capable of blocking the expression of certain pro-inflammatory molecules such as IL-1β and IL-6, thus inhibiting some inflammatory pathways and reducing photoreceptor cell death mediated by microglial activation [115].

Suppression of microglia activation using tamoxifen or a combination of tamoxifen with liposomal clodronate increases the viability and function of photoreceptor cells in the rat model mutMerTK-RP [116].

Apigenin is a flavonoid compound capable of reducing the expression of inflammatory cytokines in the retina through inhibition of microglia activation and Müller cells in *rd1* mice, favoring the survival of photoreceptors [117].

Alpha-1 antitrypsin (ATT) treatment, an immunomodulating agent, attenuates neuroinflammation by changing the polarization from microglia M1 to the anti-inflammatory phenotype M2. Thus, ATT improves visual function and reduces the degeneration of photoreceptors in *rd1* mice [118].

#### 4.1.3. Other Anti-Inflammatory Strategies

In addition to blocking TNFα or microglia activation, there are other therapeutic approaches targeting the inflammation during IRDs, especially for RP, as shown in Table 1. In recent years, treatments based on steroid hormones have been highly studied. For instance, administration of progesterone or norgestrel (a progesterone analogue) decreases photoreceptor cell death, reactive gliosis, and microglia activation and reduces the release of inflammatory factors in *rd10* mice [105,106]. Treatment with dexamethasone also decreases inflammation, contributes to cone survival, and improves visual acuity in the same murine model of RP [108]. The insertion of an intraocular implant of dexamethasone also improved visual acuity for 6 months in a patient with RP and macular edema. Thus, this suggests that numerous interventions would be required to maintain its effect over time [119].

Tauroursodeoxycholic acid (TUDCA), a main derivative of bile acid, ameliorates photoreceptor cell death in *rd10* mice, P23H rats, and S33ter-3 rats, but it fails to rescue photoreceptor cells in other RP models such as *rd1* or *rd16* mice (models with very rapid degeneration). TUDCA also shows a beneficial effect reducing cell death, inflammation, and microglia activation in mice treated with N-methyl-N-nitrosourea (MNU) or in Rpgr conditional knockout mice, other RP models [120].

Oxidative stress and inflammation are closely related pathophysiological processes. Therefore, antioxidants like lipoic acid [60,121], N-acetylcysteine [122], resveratrol [115], and *Lycium barbarum* (Goji berries) [123] can act as anti-inflammatory compounds. They reduce retinal degeneration in different murine models of RP. In RP patients, oral N-acetylcysteine administration is well tolerated, and suboptimally improves the function of macular cones [124]. Other recent clinical trials suggested that the consumption of Goji berries prevents the thinning of the macular layer in RP patients. However, this treatment does not seem to improve visual field or electrophysiological recordings [113].

Curcumin, the active ingredient in the spice turmeric, has neuroprotective effects in P23H rats and P23H swine, reducing oxidative stress in the endoplasmic reticulum (ER) and increasing rhodopsin expression [125,126]. Moreover, curcumin reduces photoreceptor cell death, microglia activation, and secretion of chemokines and matrix metalloproteinases in the retina of *rd1* mice [112].

### 4.2. “Anti-Cell Death” Therapies

Many of the above therapies aim to prevent retinal cell death. There are also therapeutic strategies focused on inhibiting apoptosis and other non-apoptotic cell death mechanisms; for instance, the gene therapy based on introducing the X-linked inhibitor of apoptosis (XIAP) protein that has neuroprotective effects on the structure and function of photoreceptors in P23H and S334ter rats [127]. Calpain inhibitors (an apoptosis pathway-mediating protein) also favor photoreceptor survival. Some of these inhibitors induce short-term neuroprotective effects but prolonged administration is associated with toxicity. Another inhibitor called calpastatin is capable of reducing photoreceptor cell death after prolonged intravitreal exposure in *rd1* mice [128].

The pharmacological inhibition of different players involved in non-apoptotic cell death mechanisms such as RIPK3 [55,77], RIPK1 [129,130], or PARP [131,132] also reduces retinal degeneration. For instance, olaparib, a PARP inhibitor, rescues photoreceptor cells in retinal explants of *rd1* mice. In vivo olaparib also decreases PARylation levels in photoreceptor cells and their cell death in *rd1* mice [132]. These results corroborate the involvement of non-apoptotic cell death mechanisms in IRDs.

Apart from the abovementioned compounds, there are many other pharmacological strategies aimed at preventing retinal degeneration during IRDs, including histone deacetylases (HDAC) inhibition [133], autophagy inhibition [134], and inhibition of ER stress [135], among others.

## 5. Conclusions and Future Perspectives

**Figure 2:**
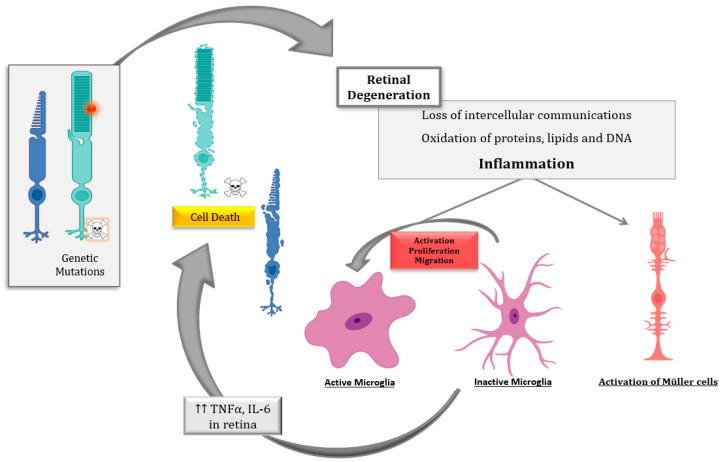
Schematic representation of the inflammatory processes underlying photoreceptor degeneration in retinitis pigmentosa, the most common inherited retinal dystrophy (IRD). The genetic defect leads to rod degeneration. During rod degeneration several cellular processes are activated, including inflammation and oxidative stress, which lead to the loss of intercellular communications, the oxidation of macromolecules, and the activation of microglia and Müller cells, among others. Activated microglia cells release inflammatory molecules such as cytokines (tumor necrosis factor alpha (TNFα) and interleukin 6 (IL6)), chemokines, etc., which would exacerbate photoreceptor degeneration (both rods and cones) through different cell death mechanism such as necroptosis, parthanatos, apoptosis, etc.

The inflammatory processes and the putative cell death mechanisms related to inflammation during IRDs, mainly RP, were reviewed. The main drivers of the death of photoreceptor (or RPE) cells in IRDs depend on the genetic defect, the cell type, and the stage of the disease. However, there are other underlying processes that contribute to the progression of retinal degeneration, including inflammation, oxidative stress, dysregulation of cGMP, intracellular calcium accumulation, or ER stress, among others [6]. Several pieces of evidence propose that inflammation is critical to both the development and exacerbation of IRDs (Figure 2).

Despite the advances in the knowledge of the cell death mechanisms involved in IRDs, the diversity of the results highlights the great complexity underlying IRDs. Several cell death mechanisms including apoptosis, necroptosis, pyroptosis, and parthanatos, among others, may be involved in photoreceptor degeneration. Inflammation and cell death are tightly interconnected and both influence each other. Therefore, the development of therapies based on ameliorating the inflammatory processes and associated cell mechanisms is crucial for slowing down IRDs. These therapies could be promising mutation-independent strategies.

## Figures and Tables

**Table 1:** Different anti-inflammatory approaches in IRDs.

Compound	Effect	Model of Degeneration or Patients	Disease	References
Adalimumab	Reduction of photoreceptor cell death, NLRP3 inflammasome components, PARP activation and microglial activation.	*rd10* mice	RP	[28]
Steroid hormones Progesterone or norgestrel	Decrease of rod and cone cell death, reactive gliosis, Activation of microglia, and release of inflammatory factors.	*rd10* mice	RP	[105,106,107]
Dexamethasone	Decrease of inflammation, increase of cone survival, and improvement of visual acuity.	*rd10* mice	RP	[108]
TUDCA	Reduction of photoreceptor degeneration, inflammation, and microglia activation, and improvement of visual function.	*Rpgr* knockout mice; mice with N-methyl-N-nitrosourea	RP, retinal degeneration	[109]
Lipoic acid	Decrease of photoreceptor cell death and microglial activation.	*rd1* mice	RP	[110]
N-acetylcysteine	Reduction in cone cell death and improvement in cone function Suppression of inflammatory factors and microglial activation.	*rd1* and *rd10* mice RP patients	RP	[111]
Turmeric (curcumin)	Decreased number of apoptotic cells, improvement of visual function, inhibition of microglial activation, and secretion of chemokines.	*rd1* mice, *661W* and BV2 cells	RP	[112]
*Lycium barbarum* (Goji berries)	Promotion of photoreceptor survival, improvement of retinal morphology, improvement in visual function; reduction of oxidative stress through the regulation of antioxidant genes.	*rd1* mice *RP* patients	RP	[113,114]
Resveratrol	Blockade of expression of pro-inflammatory molecules (IL-1β and IL-6) and protection against photoreceptor apoptosis	*661W* and *BV2* cells	Retinal degeneration	[115]
Tamoxifen and liposomal clodronate	Reduction of photoreceptor death by suppressing microglia activation.	*RCS mutMerTK-RP* rat	RP	[116]
Minocycline	Reduction of photoreceptor cell death and improvement of retinal structure and function.	*rd10* mice	RP	[45]
Apigenin	Reduction of expression of inflammatory chemokines and oxidative stress; suppression of microglia and Müller glia activation; increase in the thickness of the photoreceptor layer.	*rd1* mice	RP	[117]
Alpha-1 antitrypsin (AAT)	Attenuation of neuroinflammation by improving visual function and alleviating photoreceptor degeneration.	*rd1* mice	RP	[117]

## Data Availability

Not applicable.

## Abbreviations

AAT	Alpha-1 antitrypsin
ADA	Adalimumab
ADP-ribose	Adenosine diphosphate ribose
AIF	Apoptosis-inducing factor
AMD	Age-related macular degeneration
cGMP	Cyclic guanosine monophosphate
CNS	Central nervous system
CPR	C-reactive protein
DAMPs	Damage-associated molecular patterns
DR	Diabetic retinopathy
FADD	Fas-associated protein with death domain
GM-CSF	Granulocyte monocyte colony-stimulating factor
GSDMD	Gasdermin D
HMGB1	High mobility group box 1
IFN-γ	Gamma interferon
IL	Interleukin
iNOS	inducible nitric oxide synthase
IRDs	Inherited retinal dystrophies
JAK-STAT	Janus kinase/signal transducer and activator of transcription
M1	Pro-inflammatory microglia
M2	Anti-inflammatory microglia
MCP-1	Monocyte chemoattractant protein 1
MCP-2	Monocyte chemoattractant protein 2
MIF	Migration inhibitor factor
MAPK	Mitogen-Activated Protein Kinases
MLKL	Pseudokinase kinase-like domain of mixed-lineage kinase domain-like
NAD+	Nicotinamide adenine dinucleotide
NF-κB	Nuclear factor-κappa B
ONL	Outer nuclear layer
P	Postnatal
PAMPs	Pathogen-associated molecular patterns
PAR	Poly-ADP ribose
PARP	Poly-ADP ribose polymerase
PGF	Platelet growth factor
RANTES	Regulated upon activation, normal T cell expressed, and secreted
RIPK1	Receptor-interacting serine/threonine-protein kinase 1
RIPK3	Receptor-interacting serine/threonine-protein kinase 3
ROS	Reactive oxygen species
RP	Retinitis pigmentosa
TGF-β	Transforming growth factor beta
TRAF2	TNF receptor-associated factor 2
TNFα	Tumor necrosis factor alpha
